# A case report of platypnea-orthodeoxia syndrome: A rare condition found during diagnostic workup of a patient with embolic stroke of undetermined sources

**DOI:** 10.1016/j.ensci.2022.100393

**Published:** 2022-02-12

**Authors:** Keita Mizuma, Azusa Sugimoto, Yasuhide Mochizuki, Toshiro Shinke, Kenjiro Ono

**Affiliations:** a Division of Neurology, Department of Medicine, Showa University School of Medicine, 1-5-8 Hatanodai Shinagawa-ku, Tokyo 142-8666, Japan; b Division of Cardiology, Department of Medicine, Showa University School of Medicine, 1-5-8 Hatanodai Shinagawa-ku, Tokyo 142-8666, Japan; c Department of Neurology and Neurobiology of Aging, Kanazawa University Graduate, School of Medical Sciences, Japan

**Keywords:** Platypnea-orthodeoxia syndrome (POS), Embolic stroke of undetermined sources (ESUS), Emergency foramen ovale closure, Interatrial shunt, Transcranial color flow imaging (TC-CFI), ESUS, embolic stroke of undetermined sources, POS, platypnea-orthodeoxia syndrome, PFO, patent foramen ovale, TC-CFI, transcranial color flow imaging, TEE, transesophageal echocardiography

## Abstract

Platypnea-orthodeoxia syndrome (POS) is a rare condition wherein the magnitude of the interatrial shunt changes between the sitting and supine positions. We diagnosed POS in a case initially considered to be of embolic stroke of undetermined source and performed emergency foramen ovale closure as definitive management for the patient. In this case, additional risk factors for POS include spinal deformity, meandering of the aorta, and exclusion of the right atrium due to overextension. Transcranial color flow imaging is recommended for the diagnosis of POS because of its sensitivity, specificity, and non-invasiveness. Although POS is an important barrier to effective rehabilitation, early diagnosis and definitive management lead to dramatic clinical improvement.

## Highlights

• In POS, the magnitude of the interatrial shunt changes during repositioning.
• TC-CFI is recommended for diagnosing POS due to its sensitivity and specificity.
• Early diagnosis and definitive management lead to dramatic clinical improvement.

## 1 Introduction

Platypnea-orthodeoxia syndrome (POS) was first reported in 1949 [1]. POS is a rare condition wherein the magnitude of the interatrial shunt changes between the sitting and supine positions, and interatrial shunt due to an atrial septal defect patent foramen ovale (PFO) is the most common factor [2]. POS develops when the interatrial shunt extends horizontally due to atrial septal deformity during repositioning, making it easier for blood flow from the inferior vena cava to reach the left atrium directly [3]. We diagnosed POS in a case initially considered to be of embolic stroke of undetermined source (ESUS) and performed emergency foramen ovale closure as definitive management for the patient.

## 2 Case report

The patient was an 89-year-old right-handed woman. Her medical history included Alzheimer's disease and hypertension. The patient developed sudden right-sided paralysis and aphasia. The NIH Stroke Scale (NIHSS) score was 28 points, and brain magnetic resonance imaging revealed left middle cerebral artery obstruction. After the administration of recombinant tissue-type plasminogen activator (0.6 mg/kg), reperfusion was confirmed on the next day, and the NIHSS score improved to 0 points.

Three-dimensional computed tomography (CT) angiography, carotid artery echocardiography, transthoracic echocardiography, and Holter electrocardiography were performed to identify possible sources of the embolization; however, none were revealed. Therefore, ESUS was diagnosed. Contrast-enhanced CT showed no malignant disease or arteriosclerotic lesions in the aortic arch. Therefore, we planned to evaluate a possible right-to-left shunt using transesophageal echocardiography (TEE).

However, when the patient assumed a seated position during procedure, the patient developed rapid onset, worsening hypoxemia (the SpO2 dropped from 97% to 85%). Progression of anemia due to iliopsoas hematoma (Hb 9.4 g/dL), pneumonia, and pleural effusion were considered possible causes. Hypoxemia was improved by supine positioning, blood transfusion, diuretics, and antibiotics (SpO_2_ 95%). However, as procedure resumed, her hypoxemia recurred (SpO_2_ 88%).

**Fig. 1:**
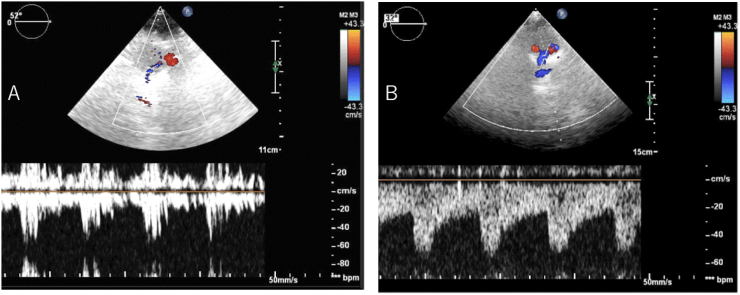
Transcranial color flow imaging (TC-CFI). Microembolic signal (MES) found in the microbubble test (right middle cerebral artery) using TC-CFI (A). Increased shunt blood flow signal intensity was observed in the sitting position. After surgery, MES disappeared in the microbubble test (B).

**Fig. 2:**
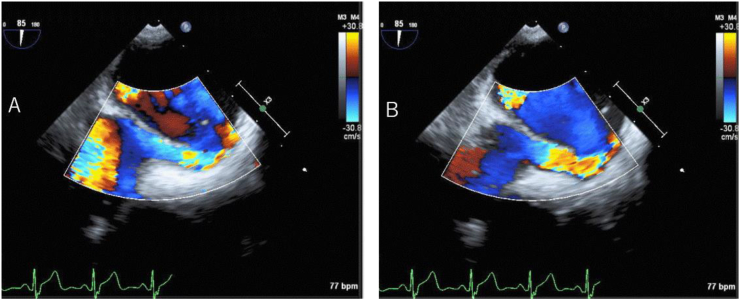
Transesophageal echocardiography (TEE). During TEE, the posture was changed from the supine position (A) to the sitting position (B).

Due to hypoxemia, a microbubble test using transcranial color flow imaging (TC-CFI) was performed prior to TEE. Marked microembolic signal (MES) of grade III (curtain pattern) was detected, with and without Valsalva loading (Fig. 1A). POS was suspected due to worsening hypoxemia in the sitting position compared to the supine position, with a marked right-to-left shunt. TEE was performed under oxygen administration, and a macroscopic PFO and atrial aneurysm were noted (Tunnel height 5.7 mm, Tunnel length 11.4 mm, Amplitude of atrial septal aneurysm 11.0 mm) (Fig. 2A). Furthermore, when the patient was repositioned from the supine position to the sitting position during the procedure, the change in echo intensity suggested an increase in shunt blood flow through the PFO, associated with worsening hypoxemia (SpO_2_ 95% to 85%) (Fig. 2B).

Deep vein thrombosis was also confirmed, and PFO-induced paradoxical embolism and POS were diagnosed. We planned elective surgical closure of the PFO, with appropriate venous thrombo-embolic prophylaxis (edoxaban 30 mg/day). However, due to respiratory distress and further exacerbation of hypoxemia (SpO_2_ 80%), emergency foramen ovale closure was performed. Immediately after the operation, the hypoxemia subsided the SpO2 has increased to 100%, and the MES disappeared in the microbubble test using TC-CFI (Fig. 1B). Following surgery, the patient was transferred to a rehabilitation hospital.

## 3 Discussion

POS was first reported in 1949 [1], and interatrial shunt due to an atrial septal defect (PFO) is the most common factor [2]. POS is a rare condition wherein the magnitude of the interatrial shunt changes between the sitting and supine positions, and there have been few reports of neurologists diagnosing POS during acute stroke management in Japan. POS develops when the interatrial shunt extends horizontally due to atrial septal deformity during repositioning, making it easier for blood flow from the inferior vena cava to reach the left atrium directly [3]. Moreover, age-related enlargement and hyperextension of the ascending aorta, deformation of the atrial septum due to pericardial fluid retention, posterior spinal bay, and iliopsoas muscle atrophy are thought to be factors that make it easier for blood flow to reach the shunt [4]. TC-CFI is recommended for the diagnosis of POS because of its sensitivity, specificity, and non-invasiveness [5].

In this case, hypoxia during sitting led to the diagnosis of POS. Additional risk factors for POS include spinal deformity, meandering of the aorta, and exclusion of the right atrium due to overextension. After admission, POS might have worsened due to iliopsoas hematoma, iliopsoas atrophy associated with bed rest, and increased oxygen requirements during procedure.

Although there are some reports of PFO and POS diagnosed after cerebral infarction, there are few reports of rapid progression of POS due to hematoma associated with treatment of cerebral infarction or prolonged bed rest, as in this case. In such rapidly progressing cases, TC-CFI at the bedside is extremely simple and useful.

## 4 Conclusion

Although POS is an important barrier to effective procedure, early diagnosis and definitive management lead to dramatic clinical improvement.

## Funding

This research did not receive any specific grant from funding agencies in the public, commercial, or not-for-profit sectors.

## Declaration of Competing Interest

None.
